# A condition-aware retrieval-augmented decision support framework for tomato cultivation management

**DOI:** 10.3389/fpls.2026.1802748

**Published:** 2026-06-18

**Authors:** Yiqun Wang, Keqing Zhao, Hongda Li, Ruihuan Wang, Wenbai Chen

**Affiliations:** 1 School of Automation, Beijing Information Science & Technology University, Beijing, China; 2 Beijing Academy of Agriculture and Forestry Sciences Information Technology Research Center, Beijing, China; 3 China Agricultural University College of Information and Electrical Engineering, Beijing, China

**Keywords:** agricultural knowledge management, condition-aware retrieval, decision support system, digital agriculture, precision crop management, retrieval-augmented generation, tomato cultivation

## Abstract

Tomato cultivation management requires decisions that depend on growth stage, environment, and production scenarios. Retrieval-augmented generation (RAG) can ground large language model (LLM) outputs in external knowledge, but conventional retrieval often ignores such conditional constraints, leading to evidence that is topically relevant yet condition-inapplicable. This study develops TSCA-RAG, a condition-aware RAG framework for tomato cultivation question answering. TSCA-RAG extracts temporal, environmental, and contextual conditions from user queries using a structured label set and employs TSCAF-Retrieval, which combines semantic retrieval, BM25 keyword retrieval, and metadata-based condition retrieval through adaptive fusion and a cross-strategy consistency reward. A tomato cultivation knowledge base was constructed as fine-grained knowledge units with condition annotations and used to evaluate both retrieval and end-to-end generation. Retrieval performance was assessed using Recall@K, MRR, and NDCG@5, and answer quality was evaluated using similarity-based and rubric-based metrics under matched generation settings. On retrieval benchmarks, TSCA-RAG improves over Fine-tuned BGE-M3 with relative gains of 5.70% in Recall@1, 4.31% in Recall@5, and 4.76% in NDCG@5. In end-to-end evaluation, TSCA-RAG achieves higher Faithfulness, Correctness, and Utility, with an 11.29% increase in Utility compared with the strongest baseline RAG system. The condition extraction module attains an overall F1 of 81.8%, and a built-in confidence attenuation mechanism recovers approximately 53% of performance loss from single-dimension extraction errors. These results indicate that explicitly modeling cultivation conditions, combined with robust extraction and adaptive error mitigation, can improve evidence applicability and response usefulness for AI-assisted tomato cultivation decision support.

## 1 Introduction

Tomato cultivation is a major component of horticultural production and underpins the economic sustainability of many agricultural regions. Achieving stable yields and consistent fruit quality requires coordinated management across the entire growth cycle, from seedling establishment to harvesting. This management depends on specialized agronomic expertise, including pest and disease diagnosis, mitigation of environmental stresses, nutrient and irrigation scheduling, and the selection of appropriate cultivation practices (Shah and Wu, 2019). In practice, however, growers do not always have access to timely and actionable technical support, and conventional extension services often fail to meet the knowledge demands of modern precision agriculture (Sapkota et al., 2025). This gap motivates the development of intelligent knowledge systems to support data-informed production decisions.

Intelligent question-answering (Q&A) systems have shown promise in agricultural applications, yet many existing platforms remain constrained by incomplete knowledge coverage and limited domain specificity (Yang et al., 2024). The rapid emergence of large language models (LLMs) further expands the technical landscape for agricultural intelligence (Vizniuk et al., 2025). However, general-purpose LLMs may be unreliable in specialized agronomic scenarios due to insufficient domain grounding, vulnerability to factual errors, and limited ability to incorporate updated technical information (Shaikh et al., 2025). These limitations are particularly consequential in tomato cultivation, where recommendations must be precise and explicitly conditioned on cultivation context.

Retrieval-augmented generation (RAG) incorporates external knowledge retrieval into response generation and has been shown to reduce hallucination and improve factual grounding (Lewis et al., 2020). RAG-based systems have also been explored in other knowledge-intensive domains (Cheng et al., 2025). Yet, directly transferring conventional RAG pipelines to agriculture remains challenging because agronomic knowledge is inherently condition-dependent: the same practice can be appropriate only under specific growth stages, environments, or management objectives (Sharmiladevi and Ravikumar, 2021). For instance, irrigation strategies differ between seedling and fruiting stages (Li et al., 2019), and disease management differs between greenhouse and open-field production systems (Panno et al., 2021). Conventional RAG retrieval, which often relies primarily on semantic similarity, may therefore return evidence that is topically related but not applicable to the user’s specific decision conditions.

To address this issue, we propose TSCA-RAG, a tomato-cultivation stage and condition-aware RAG framework for agronomic Q&A. TSCA-RAG introduces a TSCAF-Retrieval module that extracts key cultivation conditions from user queries and aligns them with structured condition metadata associated with each knowledge unit during retrieval. By combining semantically guided, fine-grained knowledge unit construction, domain-adapted embedding optimization, and condition-aware retrieval and fusion, TSCA-RAG aims to improve evidence relevance and applicability and thereby enhance the quality and consistency of generated answers.

The main contributions of this study are summarized as follows:

1. We develop a semantically guided, fine-grained knowledge unit construction approach to preserve semantic completeness and contextual coherence of agronomic knowledge.
2. We design a TSCAF-Retrieval module to explicitly model and match condition dependencies during RAG-based agricultural knowledge retrieval.
3. We implement TSCA-RAG with domain-specific embedding optimization and evaluate its effectiveness on retrieval and end-to-end tomato cultivation decision-support tasks, including a standalone assessment of the condition extraction module with error propagation analysis and system latency profiling.

## 2 Related work

### 2.1 Current status and challenges of agricultural intelligent Q&A systems

Agricultural intelligent Q&A systems have progressed from early rule-based expert systems toward data-driven platforms empowered by deep learning (Isinkaye et al., 2024). Early-generation systems were typically implemented with handcrafted expert rules or knowledge graph–based frameworks, enabling applications such as crop disease diagnosis and technical consultation (Balleda et al., 2014; Gong and Li, 2025). While such systems can be highly accurate within narrowly defined problem settings, they often depend on costly knowledge acquisition and require continuous manual maintenance as agronomic knowledge and field practices evolve. In particular, knowledge graph–based approaches are effective for entity- and relation-centric queries, yet they commonly face challenges in automated construction, scalability across diverse subdomains, and capturing deeper semantic and procedural knowledge.

With the maturation of deep learning, agricultural Q&A increasingly leverages pretrained language models and attention-based architectures. For example, BERT has demonstrated strong performance in agricultural text understanding and literature-oriented Q&A tasks (Huang et al., 2023), and attention mechanisms can facilitate the integration of information from multiple sources. Nevertheless, several persistent bottlenecks remain:

- knowledge is often represented at a coarse granularity, limiting the modeling of fine-grained features and condition dependencies;
- models may exhibit insufficient semantic understanding of specialized agronomic terminology and implicit conceptual relations;
- mechanisms for dynamic knowledge updating are frequently underdeveloped, restricting the incorporation of newly published evidence and rapidly evolving field practices. Collectively, these limitations motivate retrieval and reasoning frameworks that can better preserve knowledge integrity while adapting responses to specific cultivation conditions.

### 2.2 LLMs and prompt engineering

LLMs have achieved substantial advances in natural language understanding and generation, primarily driven by large-scale unsupervised pretraining. Their zero-shot (Gruver et al., 2023) and few-shot (Li et al., 2024) capabilities provide a foundation for interactive agricultural Q&A, enabling models to interpret natural user queries and produce fluent, coherent responses that are attractive for knowledge service delivery.

Despite these strengths, general-purpose LLMs can be unreliable in specialized agricultural settings. A major concern is hallucination, where generated outputs may appear plausible yet be factually incorrect—an especially critical risk when agronomic guidance must be precise and actionable (Tzachor et al., 2023). Moreover, the temporal cutoff of pretraining corpora can result in recommendations that lag behind updated research findings and evolving field practices (Raiaan et al., 2024). LLMs may also lack sufficient depth in agronomic terminology, implicit causal relations, and practitioner knowledge, which can reduce their utility for professional decision-making scenarios that require traceable evidence and context-sensitive constraints.

Prompt engineering is commonly adopted to steer model behavior through structured instructions or templates (Marvin et al., 2024). For instance, chain-of-thought prompting can encourage stepwise reasoning (Zhang et al., 2025b), and in-context learning can help control response structure and output style. However, prompt design in agriculture remains challenging because agronomic concepts are highly specialized, production scenarios are heterogeneous, and many management recommendations are governed by strict condition constraints (e.g., growth stage, environment, and operational objectives). Consequently, prompting alone may improve readability or reasoning style, but it does not inherently guarantee factual grounding or condition applicability. This limitation highlights the need for mechanisms that integrate external evidence and explicitly model cultivation conditions.

### 2.3 RAG paradigm and its challenges in agricultural domain

RAG aims to improve LLM reliability by integrating external knowledge retrieval into the generation process, thereby reducing hallucination and alleviating the impact of outdated parametric knowledge. A typical RAG pipeline includes three core stages: indexing (organizing and storing knowledge), retrieval (selecting relevant evidence), and generation (producing responses grounded in retrieved content). RAG-based methods have demonstrated effectiveness in several knowledge-intensive domains, including legal consultation (Hindi et al., 2025), medical decision support (Amugongo et al., 2025), and financial analysis (Li et al., 2023).

However, directly deploying conventional RAG frameworks in agricultural decision-support settings presents domain-specific challenges. First, agricultural knowledge is often procedural, hierarchical, and context-rich, making semantic completeness a central issue for knowledge unit construction (Gao et al., 2025b). For example, operational guidelines or disease management descriptions may span multiple paragraphs, and fixed-length text chunking can fragment key dependencies and disrupt semantic integrity (Yin et al., 2025). Overly coarse segmentation may introduce redundancy and dilute retrieval specificity, whereas overly fine-grained segmentation can omit essential context needed for correct interpretation. Second, general-purpose embedding models may perform suboptimally on specialized agronomic terminology and implicit domain relations (Sharma et al., 2025), degrading retrieval precision and recall in professional agronomic queries. Third, and most critically, agronomic recommendations are inherently condition-dependent: the applicability of a practice is tightly coupled with factors such as growth stage, cultivation environment, and management objectives (Young et al., 2021). Conventional RAG pipelines often rely heavily on semantic similarity and lack explicit mechanisms to represent and match these conditions. As a result, the retriever may surface evidence that is topically relevant yet condition-inapplicable, which can propagate into the generated response and reduce decision usefulness. This limitation motivates condition-aware retrieval strategies that can align query-specific cultivation constraints with structured metadata during evidence selection and fusion.

## 3 TSCA-RAG system design and implementation

**Figure 1:**
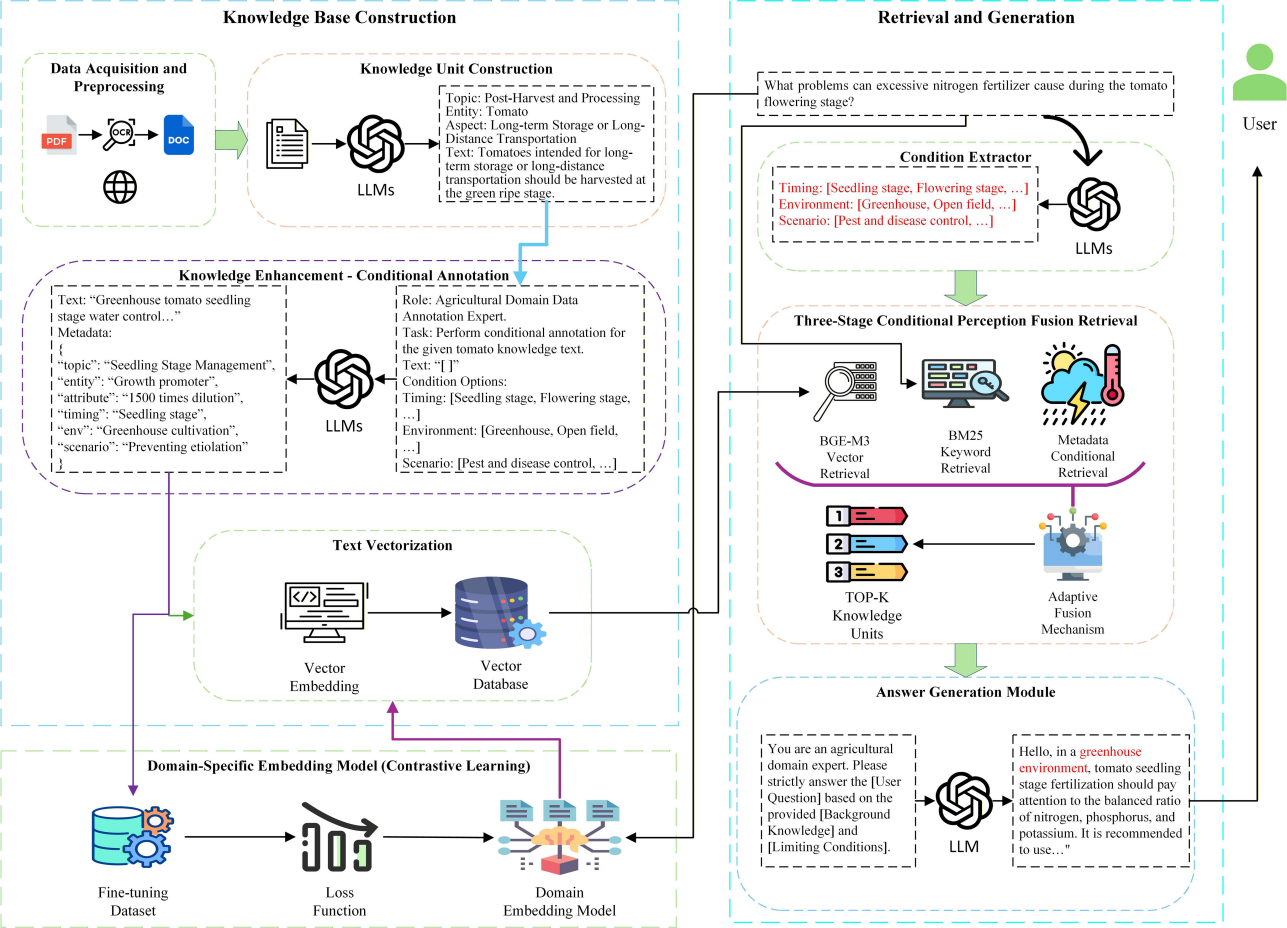
TSCA-RAG system architecture. The left part illustrates knowledge base construction: heterogeneous agricultural resources are collected and preprocessed; large language models are used to construct knowledge units; each unit is enhanced with conditional annotations to form structured metadata; and the text is vectorized and indexed in a vector database. A domain-specific embedding model is obtained via contrastive learning to improve retrieval in the tomato-cultivation domain. The right part illustrates retrieval and generation: the user query is analyzed by a condition extractor to obtain key conditions; a three-stage conditional perception fusion retrieval combines dense vector retrieval, keyword retrieval, and metadata-based conditional retrieval; an adaptive fusion mechanism ranks the most relevant knowledge units; and an answer generator produces a response grounded in retrieved evidence under the extracted conditions.

To address key limitations of conventional RAG systems in agricultural applications—coarse-grained knowledge representation, inadequate modeling of conditional dependencies, and limited domain adaptability—this study proposes the TSCA-RAG system. TSCA-RAG adopts a modular architecture composed of four core components: (i) a knowledge base construction module, (ii) a condition extractor, (iii) a TSCAF-Retrieval module, and (iv) an answer generation module. The overall technical framework is illustrated in **Figure 1**.

TSCA-RAG operates in two phases: an offline construction phase and an online service phase. During the offline phase, the knowledge base is constructed through the collection, preprocessing, and structural annotation of raw agricultural literature. LLMs are employed to automatically extract multi-dimensional conditional information—such as topic, time, environment, and cultivation context—from source documents, which is used to generate structured metadata annotations. Based on these annotations, a contrastive learning dataset is constructed to fine-tune a general-purpose embedding model, thereby enhancing its semantic representation capability in the agricultural domain. Subsequently, a multi-dimensional hybrid indexing structure is established by jointly leveraging semantic vector representations, structured metadata, and raw textual content, resulting in the construction of a semantic vector index, a metadata index, and a keyword index. During the online service phase, when a user submits a query, the condition extractor explicitly identifies and structures implicit conditional information embedded in the query. The extracted condition set, together with the query representation, jointly drives three parallel retrieval channels within the TSCAF-Retrieval module: a metadata condition retrieval channel that performs precise condition matching over the metadata index, a keyword retrieval channel that captures lexical similarity via the keyword index, and a vector retrieval channel that explores semantic associations using the domain-adapted embedding model over the vector index. The heterogeneous results returned by these channels are integrated through an adaptive weighted fusion mechanism, in which knowledge units retrieved across multiple channels receive consistency-based enhancement. The fused results are then ranked to select the top-K most relevant knowledge units. Finally, the answer generation module produces condition-aware responses grounded in the retrieved evidence.

### 3.1 Condition-aware framework design

Agricultural knowledge is strongly dependent on application conditions. Based on an analysis of tomato cultivation practice, three condition dimensions are considered:

- Temporal conditions, including growth stages (e.g., seedling, flowering, and fruiting) and seasonal factors (e.g., spring and summer).
- Environmental conditions, covering cultivation modes (e.g., greenhouse and open-field production) as well as environmental parameters such as temperature and humidity.
- Contextual conditions, referring to agronomic scenarios such as disease prevention, nutrient management, and yield optimization.

To extract such conditional information from natural language queries, an LLM-based condition extraction mechanism is implemented. DeepSeek-R1 (Guo et al., 2025) is used as the core extractor because it supports Chinese text comprehension, long-context processing, specialized terminology recognition, and structured output generation. These capabilities support identification of both explicit and implicit conditional cues while maintaining standardized outputs.

A hierarchical prompt design strategy (**Table 1**) defines a controlled vocabulary of 57 standardized condition labels across the three dimensions. The extractor is implemented as a generative structured-output module: given a query, DeepSeek-R1 outputs applicable labels in JSON format rather than performing fixed-head multi-label classification.

**Table 1:** Condition extraction and annotation prompt design.

Component	Content
Role Definition	You are an agricultural knowledge expert specializing in tomato cultivation. Please rigorously extract structured metadata from given knowledge units.
Predefined Vocabulary	Temporal conditions (timing): spring, summer, autumn, winter, seedling stage, flowering stage, fruiting stage, maturity stage, pre-seeding, post-transplanting, post-fertilization, early disease stage, early pest stage, weed occurrence period, vigorous growth period, recovery period, harvesting period Environmental conditions (environment): greenhouse, open field, hothouse, arch shed, high temperature, low temperature, high humidity, low humidity, drought, rainy, well-ventilated, poorly ventilated, sunny, weak light, fertile soil, barren soil Contextual conditions (situation): variety selection, seedling management, planting management, growth management, pruning and suckering, flower and fruit thinning, flower and fruit preservation, improving fruit set rate, fruit development, quality improvement, yield enhancement, nutrient deficiency, disease prevention, pest control, weed control, physiological disorders, soil improvement, fertilization management, water management, environmental control, harvesting, storage and preservation, processing, no applicable conditions
Annotation Constraints	1. Select only from predefined vocabulary to ensure consistency 2. Select “no applicable conditions” for basic scientific knowledge 3. Return empty array [] when no suitable vocabulary exists

To improve robustness, we adopt a three-sample self-consistency procedure under a low decoding temperature. The confidence of each candidate label is defined as its occurrence frequency across the three samples. Labels supported by at least two samples are retained as reliable conditions, whereas labels appearing only once are treated as uncertain and down-weighted during retrieval. When mutually exclusive labels are detected within the same dimension, a secondary prompt-based verification step is triggered to resolve the ambiguity. This design combines the flexibility of LLM-based extraction with the reliability required for structured condition-aware retrieval.

### 3.2 Three-stage fusion retrieval mechanism

Given the diversity and complexity of agricultural queries, reliance on a single retrieval strategy can be insufficient. TSCA-RAG therefore integrates three complementary retrieval strategies to model query–document relevance.

The first strategy is semantic retrieval, which uses a domain-adapted BGE-M3 embedding model (Praveen Gujjar and Prasanna Kumar, 2025) to encode queries and documents into dense vector representations, with cosine similarity used to measure semantic relevance. This approach captures semantic relationships at multiple granularities and is suitable for agricultural texts with complex conceptual structures.

The second strategy is BM25 keyword retrieval (Robertson and Zaragoza, 2009), which provides lexical matching and is effective for queries involving specific entities, such as cultivar names, chemical substances, or technical parameters.

The third strategy is metadata-based condition retrieval, which matches structured condition labels extracted from queries with those annotated in knowledge units. This strategy explicitly enforces condition compatibility, reducing the likelihood of retrieving semantically related but context-inapplicable evidence.

### 3.3 Adaptive fusion strategy

Different query types depend on retrieval strategies to different degrees. Queries with explicit conditional constraints typically benefit more from condition retrieval, whereas more abstract queries can rely more on semantic similarity. A dynamic weight allocation mechanism is therefore applied based on the condition extraction results.

When no explicit conditions are detected, the weights are set to *α* = 0.05, *β* = 0.60, and *γ* = 0.35 for condition retrieval, semantic retrieval, and BM25 retrieval, respectively. For queries containing a single condition, the condition weight is increased to *α* = 0.15, with *β* = 0.55 and *γ* = 0.30. When multiple conditions are present, condition retrieval is further emphasized, with *α*=0.20, *β* = 0.50, and *γ* = 0.30.

Operationally, the adaptive weighting mechanism can be viewed as a routing strategy: as the extracted condition set becomes richer, the contribution of metadata-based condition retrieval is increased, while semantic and keyword retrieval remain as fallback channels.

The final relevance score is computed as a weighted sum of the three retrieval scores:

(1) $$S(q,d)=\alpha \cdot S_{c}(q,d)+\beta \cdot S_{v}(q,d)+\gamma \cdot S_{k}(q,d)$$

Here, *S_c_*(*q*, *d*), *S_v_*(*q*, *d*), and *S_k_*(*q*, *d*) denote the relevance scores produced by condition retrieval, semantic retrieval, and BM25 retrieval, respectively. The coefficients *α*, *β*, and *γ* sum to one and are dynamically adjusted according to query complexity.

### 3.4 Consistency reward mechanism

To further improve retrieval reliability, we introduce a consistency reward mechanism based on cross-strategy agreement. The underlying assumption is that documents ranked highly by multiple, heterogeneous retrieval strategies are more likely to be truly relevant.

The consistency reward is defined in Equation 2 by comparing the ranking positions of a document across semantic and BM25 retrieval:

(2) $$R(q,d)=\max\left(0,1-\frac{\left|r_{v}(q,d)-r_{k}(q,d)\right|}{10}\right)$$

In this formulation, *r_v_*(*q*, *d*) and *r_k_*(*q*, *d*) denote the ranking positions of document *d* under semantic and BM25 retrieval, respectively. The denominator 10 serves as a normalization factor, and the max operator prevents negative rewards.

The final document score incorporates this reward, as shown in Equation 3:

(3) $$S_{f}(q,d)=S(q,d)+0.1\cdot R(q,d)$$

Here, *S*(*q*, *d*) is the fused retrieval score from Equation 1. The scaling coefficient 0.1 controls the influence of multi-strategy consensus. This mechanism reduces the risk of erroneous rankings caused by individual retrieval strategies and improves overall robustness.

### 3.5 Answer generation module design

To ensure professional quality and contextual precision in generated responses, we design a dedicated prompt template to guide the LLM in producing condition-aware agricultural advice. The prompt structure, summarized in **Table 2**, establishes a multi-layered control framework tailored to agricultural knowledge services.

**Table 2:** Prompt design for condition-aware agricultural knowledge Q&A system.

Module	Specific content
Role & Task Definition	Agricultural knowledge Q&A expert based on RAG framework, performing condition-aware agricultural guidance tasks
Core Constraints	1.Judge relevant knowledge chunks to answer the question comprehensively 2.Integrate environmental conditions to reflect spatiotemporal specificity 3.Use professional terminology with technical accuracy
Input/Output Standards	Input: {query}, {conditions}, {context} Output: Polite greeting + structured bullet-point advice with high actionability
Reliability Mechanism	Exception handling: “Insufficient knowledge coverage, please consult a domain expert”

At the core of this design is a balance between comprehensiveness and specificity. By explicitly defining the model’s role as an agricultural expert, the system encourages authoritative and technically accurate responses while avoiding overgeneralized recommendations. The model is required to integrate multiple relevant knowledge units, incorporate extracted conditions, and employ precise domain terminology.

Standardized input–output formats promote consistent system behavior and improve user experience, while explicit boundary conditions enhance safety in practical agricultural guidance. Collectively, this answer generation design strengthens the applicability, reliability, and professionalism of TSCA-RAG in real-world agricultural decision-support scenarios.

## 4 Experimental design

### 4.1 Knowledge base and dataset construction

A domain-specific dataset was developed to support the training and evaluation of the proposed TSCA-RAG system for tomato cultivation. Dataset construction comprised two main stages: (i) development of a curated agricultural knowledge base and (ii) preparation of diversified datasets for retrieval and end-to-end evaluation.

The knowledge base was compiled from four categories of authoritative tomato-cultivation sources, including monographs and reference handbooks, peer-reviewed journal articles published between 2015 and 2024, provincial and municipal extension manuals, and validated technical bulletins and practical case reports. The source composition, dataset scale, query distribution, and annotation reliability indicators are summarized in **Table 3**.

**Table 3:** Dataset composition and annotation reliability. .

Panel	Item	Value
Dataset scale	Knowledge units	2,247
	Query–document pairs	3,354
	Retrieval queries	360
	Question–answer pairs	120
Source composition	Monographs and reference handbooks	28%
	Peer-reviewed journal articles, 2015–2024	31%
	Extension manuals	24%
	Technical bulletins and case reports	17%
Query complexity	No-condition queries	78
	Single-condition queries	164
	Multi-condition queries	118
Annotation reliability	Verification subset	300 knowledge units
	Temporal label agreement	93.5%
	Environmental label agreement	84.6%
	Contextual label agreement	87.8%
	Overall label agreement	88.6%
	Semantic completeness rate	94.0%

All documents underwent preprocessing and quality control, including OCR correction, removal of front matter and bibliographic content, deduplication, and expert factual verification. The documents were then segmented into 2,247 semantically coherent knowledge units using LLM-assisted semantic chunking. During chunking and annotation, prompts were designed to preserve the association between cultivation practices and their prerequisite temporal, environmental, and contextual conditions. The same controlled vocabulary used in the online condition extraction module was applied to annotate knowledge units, ensuring consistency between query-side and document-side condition representations.

**Figure 2:**
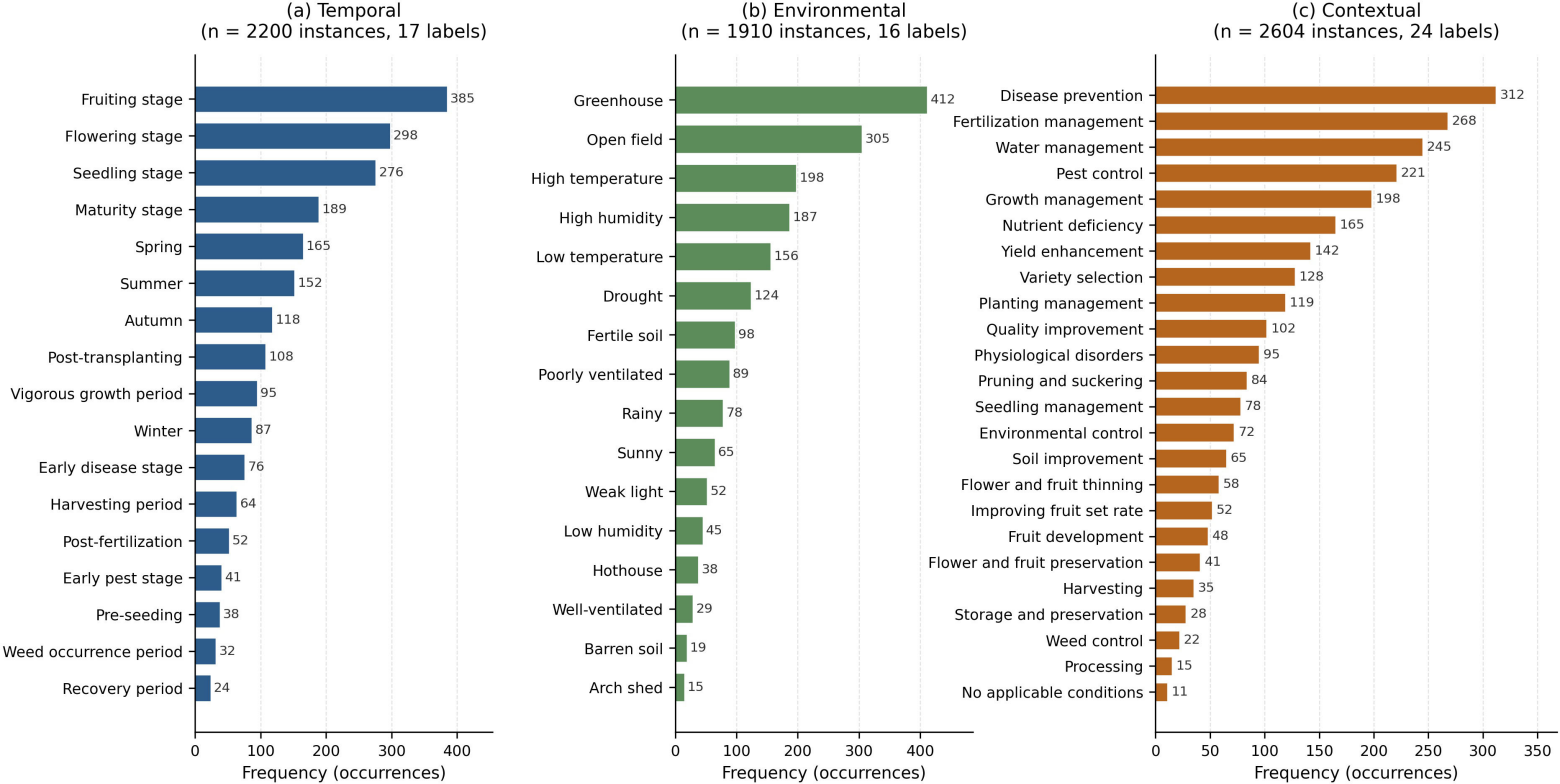
Frequency distribution of the 57 controlled-vocabulary labels across the 2,247 knowledge units, organized by condition dimension. **(a)** Temporal conditions, **(b)** Environmental conditions, **(c)** Contextual conditions. The distribution reflects typical agronomic-literature patterns, with high-frequency labels concentrated on common growth stages, cultivation environments, and routine management scenarios, while specialized or seasonal labels exhibit a long-tail distribution.

Because the annotation pipeline was based on LLM-assisted labeling followed by expert verification rather than fully independent manual annotation from scratch, LLM–expert agreement was used as the primary reliability indicator. A random subset of 300 knowledge units was independently re-examined by two domain experts, and disagreements were adjudicated by a senior agricultural specialist. The resulting agreement rates and semantic completeness rate are reported in **Table 3**. The frequency distribution of the 57 controlled-vocabulary labels is shown in **Figure 2**, where common growth stages and routine management scenarios dominate the corpus, while specialized and seasonal labels form a long-tail distribution.

For model optimization and evaluation, we constructed 3,354 query–document pairs for embedding fine-tuning, 360 retrieval queries for retrieval evaluation, and 120 question–answer pairs for end-to-end assessment. The retrieval queries include 78 no-condition queries, 164 single-condition queries, and 118 multi-condition queries, covering different levels of conditional complexity.

### 4.2 Baseline methods

#### 4.2.1 Retrieval baselines

To evaluate the effectiveness of the proposed TSCAF-Retrieval module, multiple retrieval methods were compared on the 360 test queries. Baselines were selected to represent diverse retrieval paradigms. BM25 serves as a classical sparse retrieval method with strong interpretability and robustness in keyword matching. M3E-Base (Pang et al., 2024) and BGE-M3 represent state-of-the-art general embedding models for Chinese text. A domain fine-tuned BGE-M3 model was included to assess the impact of agricultural domain adaptation. In addition, a hybrid retrieval (Omrani et al., 2024) strategy was adopted to examine the benefits of combining sparse and dense retrieval signals. The evaluated retrieval methods and their core techniques are summarized in **Table 4**.

**Table 4:** Retrieval method comparison.

Retrieval method	Core technology
BM25	Keyword matching + TF-IDF
M3E-Base	General embedding model
BGE-M3	General embedding model
Fine-tuned BGE-M3	Domain fine-tuned embedding model
Hybrid (FT BGE-M3)	Domain fine-tuning + hybrid retrieval
TSCAF-Retrieval	Three-stage condition-aware fusion retrieval

#### 4.2.2 RAG system baselines

For end-to-end evaluation, four representative RAG systems were selected as baselines. Vanilla RAG employs conventional dense semantic retrieval with a single vector representation. Hybrid RAG (Sawarkar et al., 2024) combines dense retrieval and BM25 using fixed fusion weights. DPR-style RAG (Rubin and Berant, 2024) adopts a dense passage retrieval architecture with re-ranking mechanisms, while ColBERT-style RAG (Wang et al., 2024) utilizes token-level interaction-based matching. To ensure fair comparison, all systems share the same answer generation module.

### 4.3 Evaluation metrics

To comprehensively assess system performance, both retrieval effectiveness and generated answer quality were evaluated using complementary metrics.

#### 4.3.1 Retrieval performance evaluation

Retrieval performance was evaluated based on the system’s ability to identify knowledge units that are most relevant to a given query. All metrics were computed using the annotated set of relevant knowledge units for each test query.

Recall@K (Patel et al., 2022) measures the proportion of truly relevant knowledge units retrieved by the system within the top *K* results, relative to all knowledge units relevant to the given query in the dataset. For a query *q*, let the set of truly relevant knowledge units be *G_q_*, and the top K results retrieved by the system be *R_q,k_*. Recall@K is defined in Equation 4:

(4) $$\text{Recall}@\text{K}(q)=\frac{\left|G_{q}\cap R_{q,K}\right|}{\left|G_{q}\right|}$$

The final *Recall@K* is calculated as the average across all test queries, typically for *K* values of 1, 3, and 5. Higher *Recall@K* scores indicate that the system is more effective at ranking relevant knowledge fragments among the top results.

Mean Reciprocal Rank (MRR) (Lu et al., 2023) focuses on the position where the system ranks the first correctly relevant knowledge unit. For each query *q_i_*, if its first relevant knowledge unit appears at position *rank_i_* in the retrieval result list, the reciprocal rank for this query equals $\frac{1}{{\text{rank}}_{\text{i}}}$. If no relevant knowledge unit is found within the top K retrieved results, the reciprocal rank is recorded as 0. The MRR across an evaluation set Q is calculated using Equation 5:

(5) $$\text{MRR}=\frac{1}{\left|Q\right|}\sum_{q\in Q}\frac{1}{rank_{i}}$$

MRR is particularly suitable for scenarios in which users expect to quickly locate at least one relevant answer. Higher MRR values indicate that the system is more effective in presenting the most directly relevant answers at the top of the retrieved results.

Normalized Discounted Cumulative Gain @K (NDCG@K) (Nai et al., 2024) comprehensively evaluates retrieval performance by considering both the ranking positions and the graded relevance of results. In contrast to Recall@K and MRR, which evaluate binary relevance, NDCG@K accommodates multi-level relevance assessments. It is computed in two steps. First, *DCG@K* is calculated using Equation 6:

(6) $$DCG@K=\sum_{i=1}^{K}\frac{2^{rel_{i}}-1}{\log_{2}(i+1)}$$

Where *rel_i_* represents the true graded relevance score of the result at rank *i*. To account for variations in the number of relevant documents or the distribution of relevance across queries, *DCG@K* is normalized by the ideal *DCG@K* (*IDCG@K*), as shown in Equation 7:

(7) $$NDCG@K=\frac{DCG@K}{IDCG@K}$$

*IDCG@K* is the maximum possible *DCG@K* for the given query, achieved by an ideal ranking. *NDCG@K* values range from 0 to 1, with values closer to 1 indicating that the retrieval system is effective not only in retrieving relevant results but also in ranking the most relevant documents higher, thus better fulfilling user information needs.

#### 4.3.2 Generated answer quality evaluation

Generated answers were evaluated from three complementary perspectives: semantic similarity, overall response quality, and professional accuracy.

MoverScore (Zhao et al., 2019) measures semantic similarity between generated and reference answers using contextual embeddings from pre-trained language models. Unlike traditional lexical matching approaches, MoverScore computes the optimal transport distance, known as Earth Mover’s Distance, between the distributions of the two texts in semantic space. This method can better capture synonymous expressions and conceptual substitutions, making it particularly suited for evaluating generated answers in professional domains.

LLM-based evaluation (Gao et al., 2025a) uses the closed-source language model Gemini-2.5 Pro (Comanici et al., 2025) to assess generated response quality through carefully crafted prompt templates. The model evaluates outputs across multiple dimensions, including answer accuracy, faithfulness, logical consistency, and practical utility. We adopted a 10-point scoring rubric, where a score of 1 indicates a completely inaccurate or irrelevant answer, and a score of 10 denotes a completely accurate and highly professional response. This LLM-based evaluation simulates the judgment process of human experts and provides a holistic assessment of answer quality. To improve transparency of the LLM-based scoring, the prompt design used for evaluation, including the role definition, evaluation inputs, evaluation dimensions, scoring rubric, and output format, is summarized in **Table 5**.

**Table 5:** Prompt design for LLM-based answer quality evaluation.

Component	Content
Role Definition	You are a senior agricultural expert specializing in tomato cultivation, responsible for objectively evaluating the quality of answers generated by a question-answering system.
Evaluation Input	User query, retrieved knowledge context, system-generated answer, and reference answer
Evaluation Dimensions	Faithfulness: whether the answer is grounded in the provided knowledge without fabrication; Correctness: whether the agronomic content is accurate and consistent with the reference; Utility: whether the answer is specific, actionable, and applicable to the user’s cultivation scenario
Scoring Rubric	Each dimension is rated on a 10-point scale, where 1 denotes a completely inaccurate or irrelevant answer and 10 denotes a completely accurate and highly professional response
Output Format	A score for each dimension together with a brief justification

BERTScore (Datta et al., 2022) evaluates semantic similarity based on contextualized token representations derived from pre-trained language models. The method calculates the cosine similarity between tokens of the generated text *X* and the reference text *Y*. Let *x_i_* and *y_j_* denote the contextual embeddings for tokens in *X* and *Y*, respectively. BERTScore is defined using Equations 8–10:

(8) $$R_{BERT}=\frac{1}{\left|Y\right|}\sum_{y_{j}\in Y}\underset{x_{i}\in X}{\max}x_{i}^{T}y_{j}$$

(9) $$P_{BERT}=\frac{1}{\left|X\right|}\sum_{x_{i}\in X}\underset{y_{j}\in Y}{\max}x_{i}^{T}y_{j}$$

(10) $$F1_{BERT}=2\frac{P_{BERT}\cdot R_{BERT}}{P_{BERT}+R_{BERT}}$$

This approach enables the evaluation to go beyond surface-level word matching and better capture semantic equivalence and paraphrasing.

Statistical Significance. Because all retrieval and answer-quality metrics are computed on a per-query basis, comparisons between TSCA-RAG and each baseline are treated as paired observations. Statistical significance is assessed using the two-sided Wilcoxon signed-rank test, which does not assume normality and is therefore suitable for both retrieval metrics and rubric-based scores. For each comparison, a 95% confidence interval of the mean per-query difference is estimated using a paired bootstrap with 10,000 resamples. A difference is regarded as statistically significant when the corresponding p-value is below 0.05 and the confidence interval excludes zero.

To validate the reliability of LLM-based evaluation in the agricultural domain, a partial human evaluation was conducted on a stratified 40-sample subset drawn from the 120-pair end-to-end test set, balanced across no-condition, single-condition, and multi-condition queries. Two independent evaluators with formal horticultural training and at least five years of tomato cultivation extension experience scored each answer along Faithfulness, Correctness, and Utility on the same 10-point rubric, blinded to system identity and answer order; disagreements greater than two points were adjudicated by a senior specialist. Agreement between LLM-based and human evaluations was quantified by Pearson *r*, Spearman *ρ*, and Cohen’s quadratic-weighted *κ* (after binning the 10-point scale into five intervals), with inter-human *κ* reported as a reference upper bound. Prompt sensitivity of the LLM judge was additionally assessed by re-running the scoring with two paraphrased versions of the evaluation prompt that preserved the rubric content.

## 5 Experimental results and analysis

### 5.1 Experimental setup

The semantic retrieval module employed the BAAI/bge-m3 embedding model, further fine-tuned for the tomato cultivation domain. Fine-tuning was conducted on an Ubuntu 22.04 system with an NVIDIA GeForce RTX 3090 24 GB GPU using PyTorch 2.3.0+cu121. Hyperparameters were set to a learning rate of 2e-5, batch size 16, and 8 epochs. All comparative experiments were executed under identical hardware and software settings to ensure fairness and reproducibility.

### 5.2 Retrieval module comparison results and analysis

**Table 6** reports retrieval performance on the tomato cultivation knowledge base using Recall@1/3/5, MRR, and NDCG@5. TSCA-RAG achieves the best overall performance, indicating improvements in both top-rank accuracy and ranked retrieval quality.

**Table 6:** Retrieval method performance comparison.

Model	Recall@1	Recall@3	Recall@5	MRR	NDCG@5
BM25	0.5797	0.7217	0.7855	0.6654	0.6894
M3E-Base	0.5565	0.7478	0.8029	0.6632	0.6918
BGE-M3	0.6261	0.8029	0.8609	0.7294	0.7578
Fine-tuned BGE-M3	0.6390	0.8348	0.8725	0.7386	0.7661
Hybrid (FT BGE-M3)	0.6551	0.8348	0.8957	0.7549	0.7853
TSCA-RAG	0.6754*	0.8551	0.9101*	0.7730***	0.8026***

*p< 0.05, ***p< 0.001.

Compared with Fine-tuned BGE-M3, TSCA-RAG attains a Recall@1 of 0.6754 and Recall@5 of 0.9101, corresponding to improvements of 5.70% and 4.31%, respectively. TSCA-RAG also increases MRR to 0.7730 (4.66% improvement) and NDCG@5 to 0.8026 (4.76% improvement). Paired Wilcoxon signed-rank tests confirm that the improvements over Fine-tuned BGE-M3 are statistically significant for Recall@1, Recall@5, MRR, and NDCG@5 (p< 0.05, and p< 0.001 for MRR and NDCG@5), with 95% bootstrap confidence intervals of the mean per-query difference that exclude zero. These gains suggest that incorporating explicit condition information into the retrieval process improves both the likelihood of retrieving relevant evidence within the top-K set and the prioritization of more applicable knowledge units.

Relative to Hybrid (FT BGE-M3), TSCA-RAG further improves Recall@5 by 1.61% and NDCG@5 by 2.20%. This improvement indicates that while hybrid lexical–semantic retrieval alleviates vocabulary mismatch; explicit condition modeling is more effective at reducing the retrieval of semantically similar but condition-inapplicable knowledge.

Overall, the results in **Table 6** demonstrate that TSCA-RAG achieves superior retrieval performance by jointly enhancing recall capacity and ranking robustness.

### 5.3 Retrieval module ablation study results and analysis

**Figure 3:**
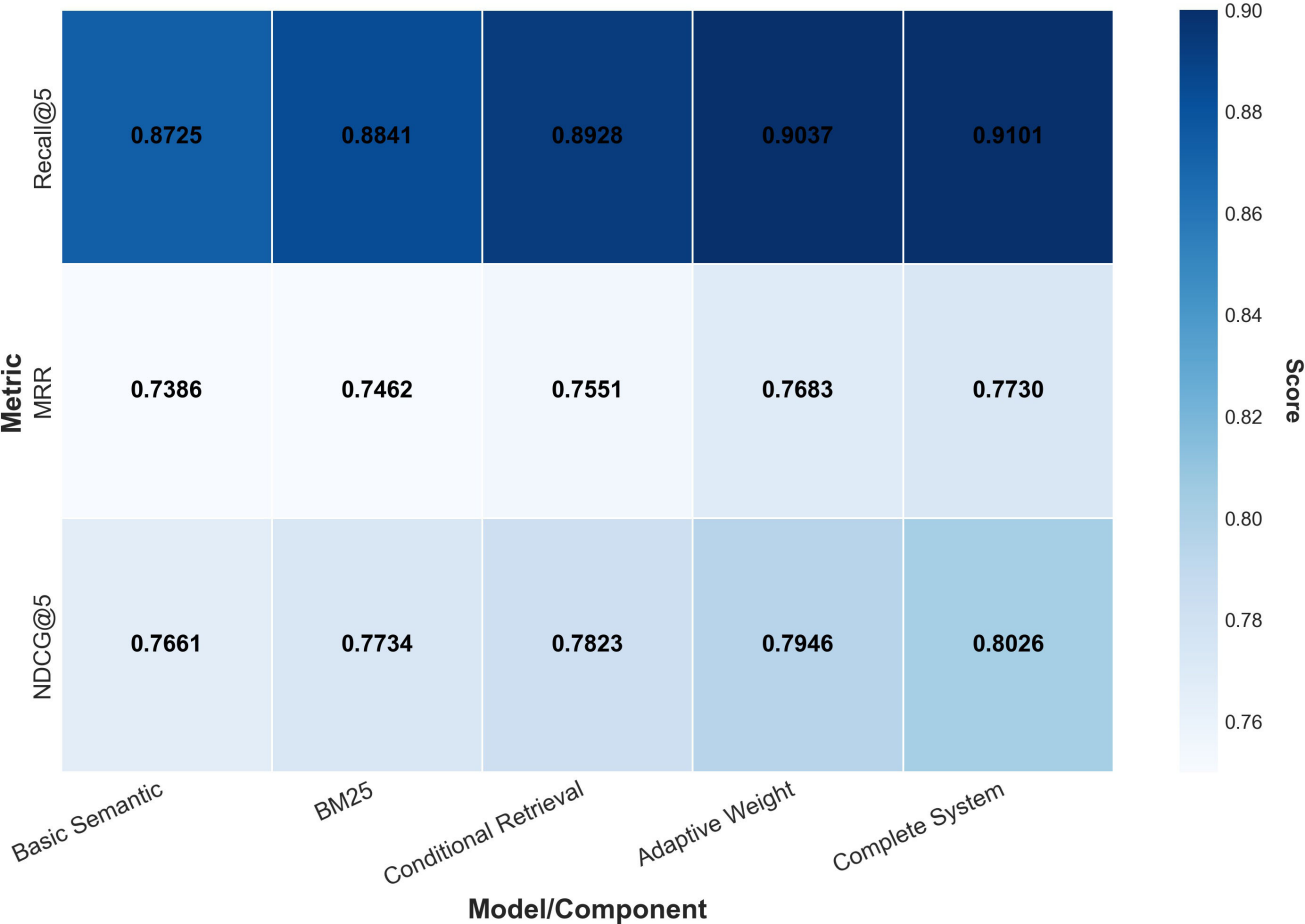
Retrieval module ablation experiment results. Heatmap reports retrieval effectiveness under different configurations. Darker shading indicates higher scores, showing that incorporating condition-aware retrieval and adaptive fusion yields the best overall performance.

**Figure 3** illustrates the ablation study results of TSCAF-Retrieval using a heatmap representation. The experiments progressively incorporate different retrieval components to quantify their individual and cumulative contributions. The analysis is organized according to the retrieval bottleneck addressed by each component and the resulting metric changes.

After incorporating BM25 as a complementary retrieval signal, Recall@5 increases from 0.8725 to 0.8841, corresponding to a 1.33% improvement. This result indicates that lexical matching remains beneficial for queries involving cultivar names, chemical substances, or highly specific agronomic terminology.

With condition retrieval enabled on top of BM25 augmentation, Recall@5 further increases to 0.8928, yielding an additional 0.98% improvement. Under the same setting, MRR rises from 0.7462 to 0.7551, representing a 2.23% improvement. These changes reflect improved applicability alignment, where retrieved evidence better matches the temporal and environmental constraints expressed in the query.

After introducing adaptive weighting, Recall@5 improves from 0.8928 to 0.9037, while MRR increases from 0.7551 to 0.7683. This trend suggests that dynamically adjusting the relative contributions of semantic, lexical, and condition-aware signals enables the retrieval module to better accommodate varying query complexities.

Finally, with the consistency reward mechanism, Recall@5 reaches 0.9101 and NDCG@5 increases to 0.8026. Compared with the baseline configuration, this represents a 4.31% improvement in Recall@5 and a 4.76% improvement in NDCG@5. These results support the intended role of the consistency reward in promoting evidence that is consistently supported across multiple retrieval strategies, thereby enhancing ranking robustness.

Overall, the complete TSCAF-Retrieval configuration demonstrates cumulative and complementary performance gains, indicating that each module contributes distinct benefits rather than redundant effects.

### 5.4 Evaluation of the condition extraction module

The overall performance of TSCA-RAG relies on the accuracy of the upstream condition extraction module. To independently assess extraction quality and quantify the impact of extraction errors on downstream retrieval, we conducted two sets of experiments: a standalone evaluation of extraction performance and a controlled error propagation analysis.

#### 5.4.1 Standalone extraction performance

**Figure 4:**
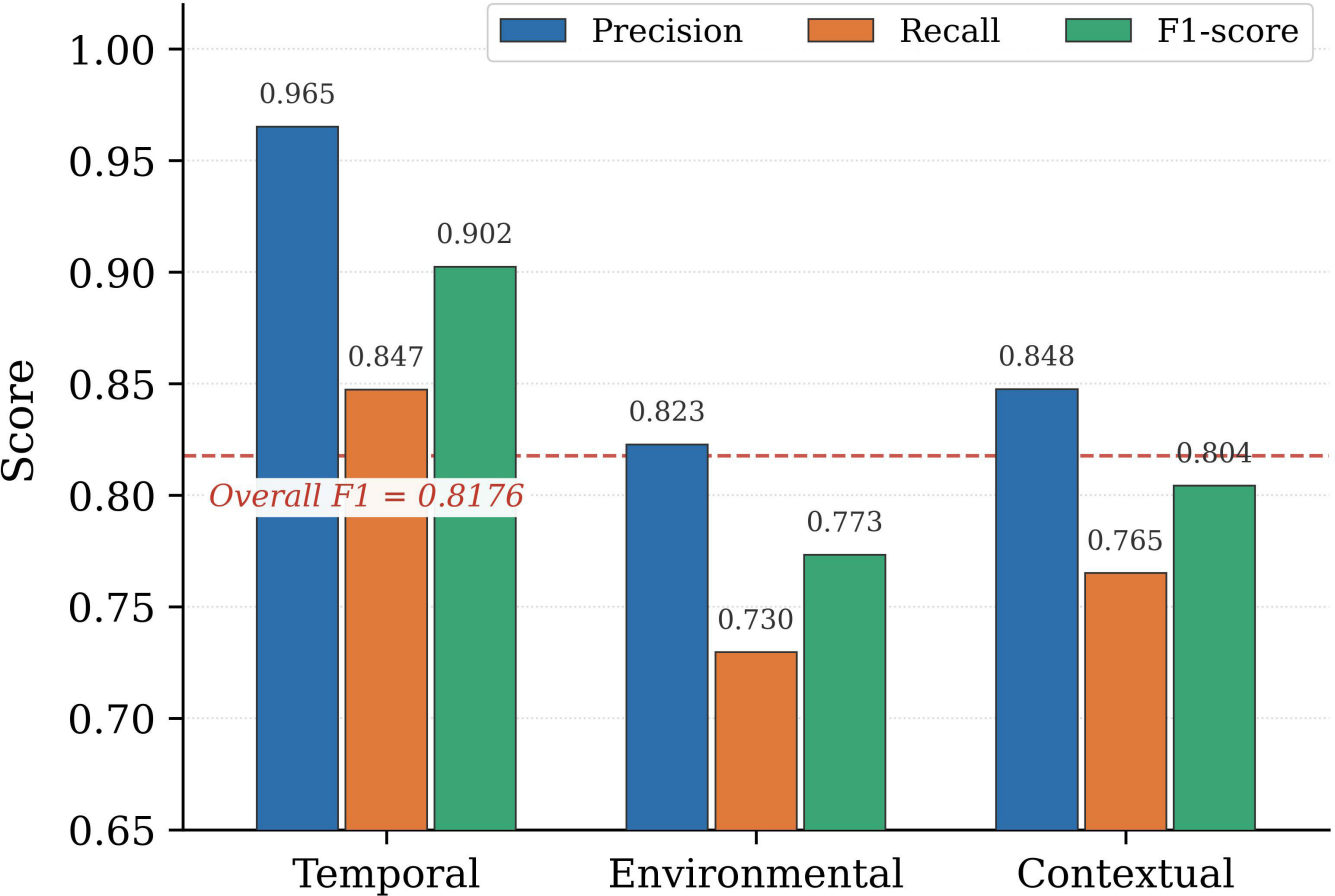
Precision, Recall, and F1-score of the condition extraction module across three condition dimensions. The dashed line indicates the overall weighted F1-score (0.8176). Temporal conditions achieve the highest performance due to explicit lexical cues, while environmental conditions exhibit the lowest scores, reflecting the difficulty of extracting implicitly expressed conditions.

A total of 200 queries were randomly sampled from the 360-query test set and manually annotated with reference condition labels according to the predefined vocabulary. The extraction module was then evaluated against these reference labels using precision, recall, and F1-score, computed at the label level across all three condition dimensions. The results are summarized in **Figure 4**.

Among the three condition dimensions, temporal conditions achieved the highest F1 (90.2%), primarily because growth stage indicators such as “seedling stage” and “fruiting stage” tend to appear as explicit lexical cues in user queries, making them relatively straightforward for the extractor to identify. Environmental conditions proved the most challenging, yielding the lowest F1 of 77.3%, approximately 13 percentage points below temporal conditions. This gap is attributable to the implicit nature of environmental cues: for instance, a query mentioning “poor ventilation leading to high humidity” implies both “poorly ventilated” and “high humidity,” yet the model occasionally failed to capture both labels simultaneously. Contextual conditions achieved an intermediate F1 of 80.4%, with errors mainly arising from overlapping management scenarios such as “fertilization management” and “nutrient deficiency,” which can co-occur within a single query but are occasionally conflated by the extractor. Across all dimensions, precision consistently exceeded recall, suggesting that the extractor is conservative in its assignments: it assigns labels with high confidence but tends to omit conditions that are not explicitly stated.

A further examination of error types revealed three dominant failure modes. Confusion between adjacent growth stages accounted for 31.4% of all extraction errors, as stages such as flowering and early fruiting share overlapping physiological descriptions. Omission of implicit environmental conditions constituted 28.6%, reinforcing the observation that environmentally embedded cues are the primary extraction bottleneck. Conflation of closely related contextual labels accounted for 22.9%. These findings indicate that future improvements should prioritize inference of implicit environmental conditions and disambiguation of semantically adjacent labels.

**Figure 5:**
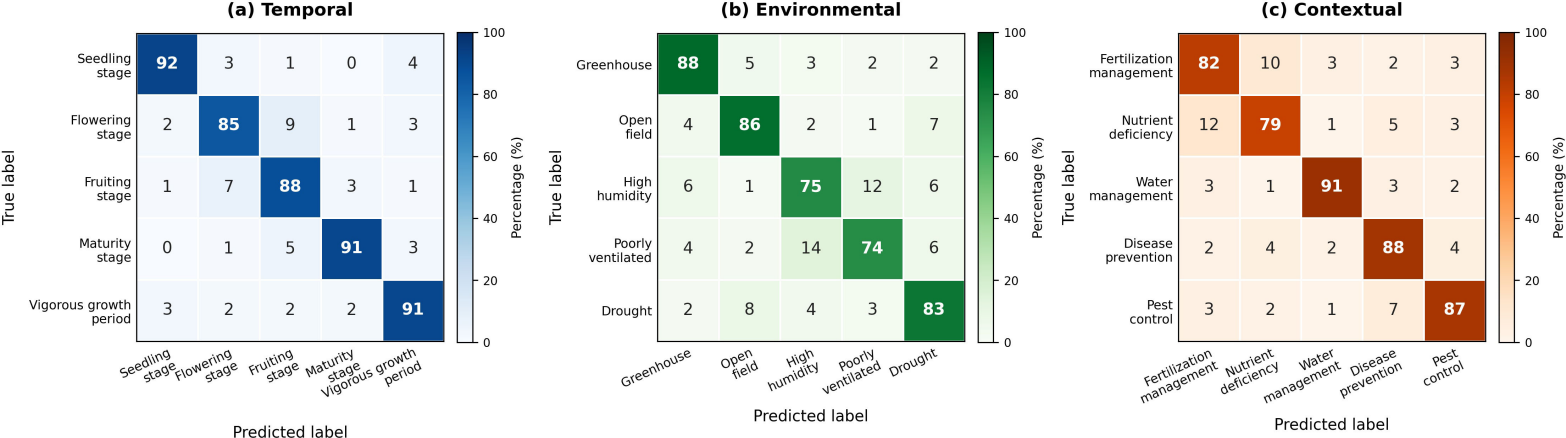
Confusion patterns of the condition extraction module on the 200-query annotated test sample, shown for the five most frequent labels in each condition dimension; values are row-normalized to percentages and diagonal cells represent correct extraction. **(a)** Temporal dimension exhibits localized confusion between adjacent growth stages (flowering stage ↔ fruiting stage). **(b)** Environmental dimension shows substantial bidirectional confusion between high humidity and poorly ventilated, reflecting the difficulty of disambiguating co-occurring implicit cues. **(c)** Contextual dimension reveals overlap between fertilization management and nutrient deficiency, which frequently co-occur in the same query context.

**Figure 5** further visualizes the dominant confusion patterns among the five most frequent labels in each condition dimension. The results confirm that extraction errors are localized rather than uniformly distributed: temporal errors mainly occur between adjacent growth stages, environmental errors arise from co-occurring implicit cues, and contextual errors are concentrated in overlapping management scenarios. These patterns provide diagnostic evidence for future improvements in implicit condition inference and label dependency modeling.

#### 5.4.2 Error propagation analysis

To quantify the downstream impact of extraction errors, a controlled perturbation experiment was conducted on 80 queries that contained at least one annotated condition. Two perturbation settings were applied: (i) single-dimension perturbation, in which one condition label was replaced with a semantically related but incorrect label within the same dimension (e.g., “flowering stage” replaced by “fruiting stage”); and (ii) multi-dimension perturbation, in which labels across two dimensions were simultaneously altered (e.g., “flowering stage” replaced by “fruiting stage” and “greenhouse” replaced by “open field”). For each setting, retrieval and end-to-end generation were re-executed, and results were compared against the correct extraction baseline. The results are presented in **Table 7**.

**Table 7:** Impact of condition extraction errors on downstream performance.

Setting	BERTScore	Faithfulness	Correctness	Utility
Correct extraction (baseline)	0.8062	9.87	9.67	9.70
Single-dimension perturbation	0.7981 (-1.01%)	7.30 (-26.0%)	6.93 (-28.3%)	6.30 (-35.1%)
Multi-dimension perturbation	0.7932 (-1.61%)	5.13 (-48.0%)	4.47 (-53.8%)	4.00 (-58.8%)
Single-dim. + confidence attenuation	**0.8015 (-0.58%)**	**8.77 (-11.1%)**	**8.27 (-14.5%)**	**8.10 (-16.5%)**

Bold values indicate the best-performing result in each column.

A notable finding across both perturbation settings is the divergence between BERTScore and LLM-based metrics. Under single-dimension perturbation, BERTScore decreased by only 1.01%, whereas Utility dropped by 35.1%. This contrast indicates that condition errors preserve surface-level textual similarity, as the retrieved evidence still covers the correct topic. However, they substantially degrade practical actionability, because condition-mismatched evidence introduces management recommendations that are inapplicable to the user’s specific scenario. Under multi-dimension perturbation, the degradation was compounding rather than additive: Utility fell by 58.8% and Correctness by 53.8%, reflecting the simultaneous loss of alignment across temporal and environmental constraints. When both condition dimensions are incorrect, the retrieval module effectively loses the ability to distinguish between knowledge units that differ only in applicability context. As a result, the generated responses conflate advice from incompatible cultivation scenarios.

The confidence-based attenuation mechanism substantially mitigated the impact of single-dimension errors, reducing the Utility degradation from 35.1% to 16.5%, which corresponds to a recovery of approximately 53% of the performance loss. This mitigation operates by downweighting uncertain condition labels and shifting retrieval emphasis toward semantic and keyword channels, which serve as fallback mechanisms when condition information is unreliable. These results confirm two key properties of the system: extraction accuracy has a direct and substantial impact on response quality, and the adaptive architecture provides meaningful built-in resilience against moderate extraction errors.

### 5.5 RAG system comparison results and analysis

Having established the standalone performance and error resilience of the condition extraction module, we now compare the complete TSCA-RAG system against baseline RAG configurations. As reported in **Table 8**, TSCA-RAG is compared with four baseline RAG systems under an identical generation configuration using DeepSeek-R1. Therefore, observed performance differences primarily reflect retrieval quality and evidence grounding capability.

**Table 8:** RAG system end-to-end performance comparison.

RAG system	Moverscore	LLM-based			BERTScore
		Faithfulness	Correctness	Utility	
Vanilla RAG	0.5534	7.01	7.71	6.74	0.7581
Hybrid RAG	0.5602	7.94	8.42	8.15	0.7759
DPR-style RAG	0.5687	7.79	8.34	7.76	0.7684
ColBERT-style RAG	0.5591	6.96	7.62	6.54	0.7703
TSCA-RAG	**0.5753 ns**	8.57**	9.16**	9.07**	0.7922*

Bold values indicate the best-performing result in each evaluation metric. *p < 0.05, **p < 0.01, ns: not significant.

On similarity-based metrics, TSCA-RAG achieves a MoverScore of 0.5753, which is 1.16% higher than the strongest baseline value of 0.5687. TSCA-RAG also attains a BERTScore of 0.7922, corresponding to a 2.10% improvement over the strongest baseline BERTScore of 0.7759. Although these gains are moderate, they are consistent across both similarity metrics. A paired significance analysis indicates that the BERTScore improvement over the strongest baseline is statistically significant (Wilcoxon signed-rank, p< 0.05, 95% CI excluding zero), whereas the smaller MoverScore difference does not reach significance, which suggests that the benefit of condition-aware retrieval is concentrated in evidence grounding rather than in surface-level similarity.

More substantial improvements are observed on LLM-based evaluation dimensions. TSCA-RAG achieves a Faithfulness score of 8.57, a Correctness score of 9.16, and a Utility score of 9.07. Relative to the strongest baseline systems, these correspond to improvements of 7.93%, 8.79%, and 11.29%, respectively. These results indicate that TSCA-RAG produces responses that are more strongly grounded in retrieved evidence and offer higher practical usefulness for cultivation decision support. The improvements on Faithfulness, Correctness, and Utility are all statistically significant (paired Wilcoxon signed-rank, p< 0.01).

It is worth noting that ColBERT-style RAG obtains a Utility score of 6.54, below the 6.74 of Vanilla RAG, even though it performs token-level interaction. A plausible explanation lies in the lexical characteristics of tomato cultivation texts: management recommendations across different growth stages and environments share a large common vocabulary, including terms such as irrigation, soil moisture, and fertilization. Token-level matching therefore tends to assign high scores to knowledge units that are lexically close but describe incompatible cultivation conditions, and the resulting evidence mixes advice from divergent scenarios. Utility is the metric most affected by this, since it reflects whether the response is directly applicable to the user’s situation. Vanilla RAG, relying on a single dense representation, is less discriminative at the token level but tends to return evidence with more consistent thematic focus. The gap between the two baselines highlights that, in this domain, lexical granularity without condition awareness can be counterproductive rather than beneficial.

### 5.6 Reliability of LLM-based evaluation

To assess whether the LLM-based scores reported in Section 5.5 are consistent with expert judgment, the five RAG systems were re-evaluated on the 40-sample stratified subset by two human experts following the protocol in Section 4.3.2. As shown in **Table 9**, the LLM judge achieved Spearman correlations of 0.74-0.81 and quadratic-weighted κ values of 0.68-0.76 with human experts, comparable to the inter-human κ range of 0.72-0.79.

**Table 9:** Agreement between LLM-based and human evaluations on a 40-sample stratified subset.

Dimension	Pearson	Spearman	Quadratic-weighted κ	Inter-human κ	LLM–Human mean offset
Faithfulness	0.71	0.74	0.68	0.72	+0.51
Correctness	0.79	0.81	0.76	0.79	+0.38
Utility	0.76	0.78	0.73	0.75	+0.36
Average	0.75	0.78	0.72	0.75	+0.42

The LLM judge showed a mild leniency bias, with an average offset of +0.42 points on the 10-point scale, but preserved the relative ranking of systems across all three dimensions. Prompt sensitivity was limited, with a mean per-sample deviation of 0.31 points across paraphrased prompt variants and unchanged system rankings. These results indicate that LLM-based evaluation is suitable for relative system-level comparison in this study, although the scores should not be interpreted as absolute measures of professional answer quality.

### 5.7 Performance comparison analysis across different generation models

To examine the robustness of TSCA-RAG across different generation backbones, we selected four representative language models for comparison: GPT-4o (Shahriar et al., 2024) represents state-of-the-art technical performance, DeepSeek-R1 excels in Chinese understanding and reasoning, Kimi-K2 (Team et al., 2025) is suitable for long text processing, and Qwen-max (Zhang et al., 2025a) demonstrates advantages in Chinese text generation. This diversified model selection ensures comprehensive and reliable evaluation results, with experimental results shown in **Table 10**.

**Table 10:** TSCA-RAG system end-to-end performance comparison across different LLMs.

LLMs	MoverScore	LLM-based			BERTScore
		Faithfulness	Correctness	Utility	
GPT-4o	0.5861	9.12	9.42	9.37	0.8161
DeepSeek-R1	0.5753	8.57	9.16	9.07	0.7922
Kimi-K2	0.5653	8.24	9.05	8.91	0.7351
Qwen-max	0.5774	8.79	9.27	9.15	0.7952

GPT-4o achieves the best overall performance. Compared with DeepSeek-R1, GPT-4o improves MoverScore by 1.88% and BERTScore by 3.02%. GPT-4o also improves Faithfulness by 6.42%, Correctness by 2.84%, and Utility by 3.31%. These results indicate that stronger generators can further enhance both evidence-aligned quality and similarity-based performance under an identical retrieval foundation.

Qwen-max yields performance close to DeepSeek-R1, with smaller but consistent gains: Faithfulness increases by 2.57%, Correctness by 1.20%, Utility by 0.88%, and BERTScore by 0.38%. In contrast, Kimi-K2 produces lower scores in this setting; relative to DeepSeek-R1, its BERTScore decreases by 7.21%, with smaller reductions in Faithfulness, Correctness, and Utility.

The low BERTScore of Kimi-K2 (0.7351) contrasts with its relatively stable Correctness and Utility scores. BERTScore is computed from token-level cosine similarity between generated and reference texts and is therefore sensitive to lexical choice, sentence length, and syntactic structure. Kimi-K2 is optimized for long-context processing and tends to produce more verbose, paraphrased responses that diverge from the reference in surface form while retaining the underlying agronomic content. LLM-based evaluation, which judges responses holistically, is more tolerant of such stylistic variation. A similar pattern can be observed across the four generators, where differences on LLM-based metrics remain small. This suggests that the retrieval evidence supplied by TSCAF-Retrieval is stable enough to anchor correctness and utility regardless of generation style, while surface-level similarity metrics remain sensitive to the individual wording habits of each model.

Overall, the comparison suggests that while TSCA-RAG provides stable retrieval evidence across generators, end-to-end performance remains sensitive to the choice of generation model.

### 5.8 Case analysis of TSCA-RAG in tomato cultivation

**Figure 6:**
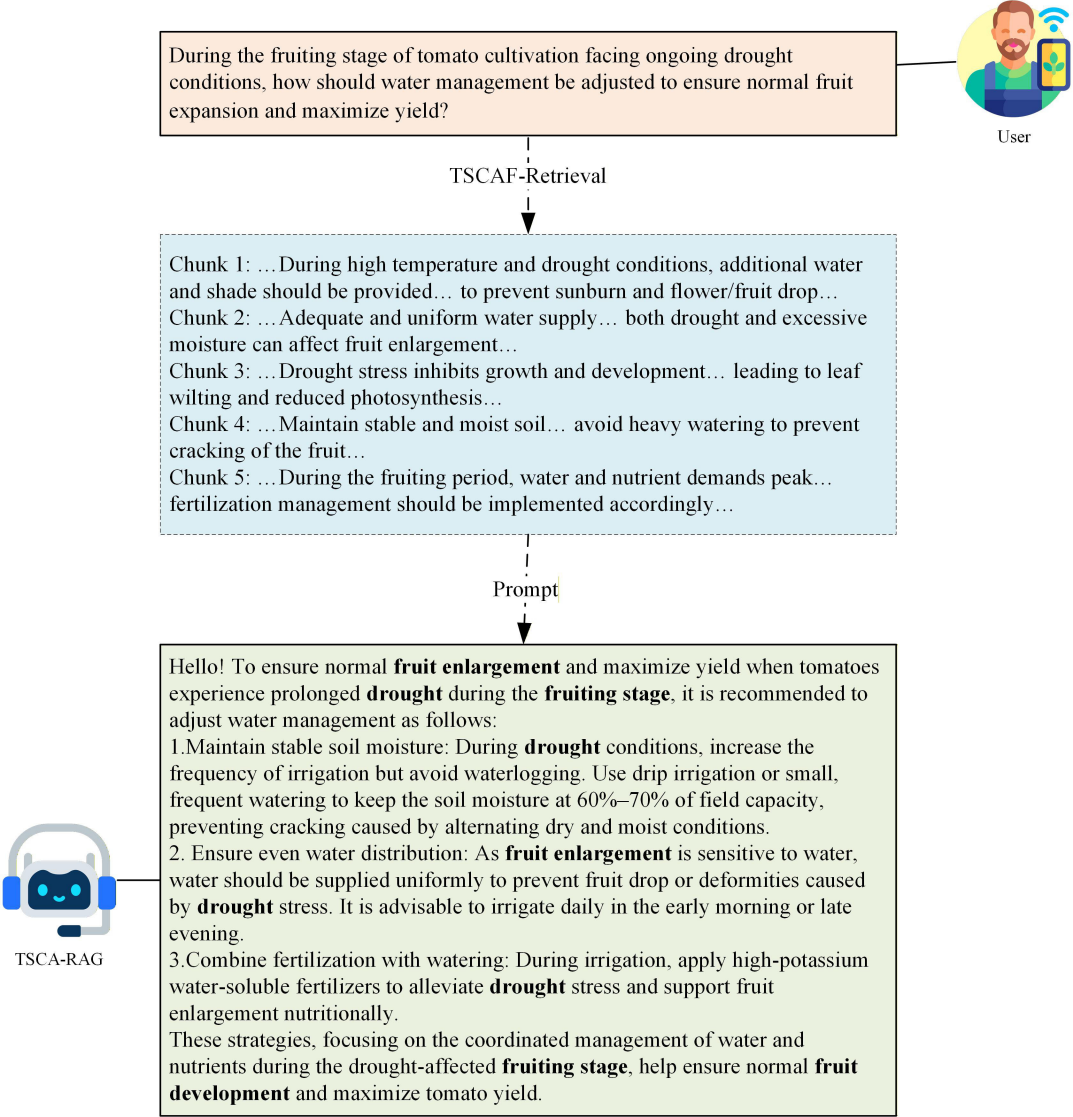
Case Study Demonstration of TSCA-RAG System Workflow for Condition-Aware Agricultural Q&A. Given a user question about adjusting irrigation during the tomato fruiting stage under drought, TSCAF-Retrieval retrieves multiple evidence chunks that match both topical relevance and conditional constraints. The final answer is generated based on the retrieved evidence and provides actionable recommendations, such as maintaining stable soil moisture with appropriate irrigation frequency, ensuring uniform water distribution to avoid fruit disorders, and coordinating fertilization with irrigation to support fruit enlargement under drought stress.

**Figure 6** presents a representative case illustrating the condition-aware retrieval and evidence-grounded generation process of TSCA-RAG. The query focuses on water management strategies during the tomato fruiting stage under sustained drought conditions.

The system first extracts salient conditions from the query, including the fruiting stage and drought stress, and retrieves knowledge units whose annotated applicability matches this combined condition set using TSCAF-Retrieval. The retrieved evidence covers irrigation frequency, soil moisture stability, drought stress mitigation, and fertilization coordination.

Based on the retrieved evidence, TSCA-RAG generates a structured and context-aligned advisory response following the predefined prompt template. The case demonstrates how explicit condition modeling constrains evidence selection and supports a more coherent and practically applicable synthesis within the same RAG pipeline.

To provide a more balanced assessment of system behavior, we further examine a failure case and a boundary condition scenario.

Failure case. One natural extraction error occurred on a query about damping-off disease in tomato seedlings grown in persistently wet soil. The stem-base softening symptoms described in the query partially overlap with descriptions of root disorders at later growth stages, and the extractor assigned “flowering stage” instead of the correct “seedling stage”. Retrieval was redirected toward flowering-stage knowledge, and the response recommended humidity control for fruit set while omitting seedling-specific interventions such as substrate sterilization and fungicide drenching. After manual correction of the stage label, the system produced an accurate, stage-appropriate answer. The case points to a structural limitation of the current extractor: labels in each condition dimension are processed independently, so the co-occurrence of “high humidity” and “disease prevention”, which together strongly imply an early-stage soil-borne pathogen scenario, cannot be fully exploited. Modeling cross-dimensional label dependencies is a natural direction for addressing such borderline queries.

Boundary case. A query asking for the most common and easiest tomato variety to grow contains no temporal or environmental cues. The extractor returned a single contextual label (“variety selection”), which activated the single-condition weight configuration, and retrieved knowledge units spanned multiple seasons, environments, and production objectives. The generated response was accurate but broadly scoped, without prioritizing any specific scenario. The behavior reflects a deliberate design choice. Treating condition absence as low confidence and falling back to semantic retrieval preserves graceful degradation on underspecified queries. It also exposes a trade-off, since the system does not actively elicit missing conditions. In an interactive deployment, a clarification step that prompts users for key conditions when extraction yields insufficient labels would narrow the retrieval scope without sacrificing the current fallback behavior.

### 5.9 System latency analysis

For practical deployment as an interactive Q&A service, system response time is a critical consideration. To evaluate the online inference efficiency of TSCA-RAG, we measured the end-to-end latency on 120 test queries using the same hardware configuration described in Section 5.1. All LLM inference calls were executed via API access to DeepSeek-R1, and latency was measured from query submission to the completion of answer generation. The results are summarized in **Table 11**.

**Table 11:** Average latency breakdown of the TSCA-RAG online pipeline.

Pipeline stage	Average latency	Proportion (%)
Condition extraction	1.66 s	12.4%
Three-stage parallel retrieval	1.35 s	10.1%
Adaptive fusion and ranking	< 0.01 s	< 0.1%
Answer generation	10.36 s	77.5%
Total	13.37 s	100.0%

The average end-to-end latency per query is 13.37 seconds. As shown in **Table 11**, answer generation accounts for 77.5% of the total latency, and together with condition extraction, the two LLM inference stages account for approximately 89.9%. In contrast, the three-stage retrieval and adaptive fusion components contribute only 1.35 seconds, indicating that the proposed condition-aware retrieval mechanism introduces limited computational overhead.

Because all condition extraction and answer generation calls were executed through the public DeepSeek-R1 API, the reported latency includes network round-trip time and server-side queuing overhead. This explains why TSCA-RAG is slower than compact locally served agricultural chatbots such as Krishi Sathi, which reports an average response time of under 6 seconds (Vijayvargia et al., 2025). Therefore, the main deployment bottleneck lies in the LLM backend rather than in the retrieval design.

Future optimization should focus on replacing the LLM-based condition extractor with a lightweight domain-fine-tuned classifier, adopting streaming generation, caching recurring query patterns, and deploying locally hosted models with optimized inference engines. Although 13.37 seconds may not satisfy highly interactive real-time chat requirements, it may remain acceptable for expert-consultation-oriented agricultural decision-support scenarios, where users expect comprehensive and evidence-grounded responses.

## 6 Conclusion

This study develops TSCA-RAG, a condition-aware retrieval-augmented generation framework for tomato cultivation Q&A. The proposed TSCAF-Retrieval module integrates semantic retrieval, BM25 keyword retrieval, and metadata-based condition retrieval with adaptive fusion and a cross-strategy consistency reward. On the tomato cultivation knowledge corpus, TSCA-RAG achieves relative gains of 5.70% in Recall@1, 4.31% in Recall@5, and 4.76% in NDCG@5 over Fine-tuned BGE-M3, along with improvements in Faithfulness, Correctness, and Utility in end-to-end evaluation compared with baseline RAG systems. The condition extraction module attains an overall F1 of 81.8%, with temporal conditions exhibiting the highest accuracy and environmental conditions presenting the greatest challenge due to implicit expression patterns. Error propagation analysis demonstrates that the confidence-based attenuation mechanism recovers approximately 53% of the performance loss from single-dimension extraction errors. System latency analysis reveals an average end-to-end response time of 13.37 seconds, of which the retrieval and fusion stages contribute only 1.35 seconds, indicating that the condition-aware retrieval mechanism introduces minimal computational overhead. Compared with metadata-aware RAG approaches outside agriculture, which commonly use document attributes such as source, time, topic, or access constraints to filter or rerank retrieved passages, TSCA-RAG treats cultivation conditions as domain applicability constraints. In this setting, metadata are not merely provenance descriptors but agronomic prerequisites that determine whether a recommendation is valid for a specific growth stage, environment, or management objective. Therefore, TSCA-RAG differs from fixed metadata filtering by adaptively fusing condition matching with semantic and lexical relevance, allowing the system to retain fallback retrieval capacity when query conditions are incomplete or uncertain.

Several limitations remain. First, environmental condition extraction remains challenging because many environmental cues are implicit, and the current extractor does not explicitly model cross-dimensional label dependencies. Second, TSCA-RAG does not actively elicit missing conditions from underspecified queries. Third, the average latency of 13.37 seconds may limit highly interactive deployment, although most of the delay originates from remote LLM inference rather than retrieval. Finally, the evaluation is restricted to tomato cultivation. Although the three condition dimensions are crop-agnostic, the controlled vocabulary is partially tomato-specific; adapting the framework to other crops would require vocabulary revision, offline re-annotation, and embedding fine-tuning. Future work will therefore focus on implicit condition inference, dependency-aware extraction, lightweight local inference, multimodal symptom inputs, and validation across additional crop domains.

## Data Availability

The raw data supporting the conclusions of this article will be made available by the authors, without undue reservation.
